# Lactating Adenoma Arising From Ovarian Mature Teratoma: An Unusual Presentation of a Young Pregnant Woman

**DOI:** 10.7759/cureus.65846

**Published:** 2024-07-31

**Authors:** Sharmilla Thirugnanasambandan, Kalaivani Amitkumar, Muthu Sudalaimuthu

**Affiliations:** 1 Department of Pathology, SRM Medical College Hospital and Research Center, SRM Institute of Science and Technology, Chengalpattu, IND

**Keywords:** choroid plexus, germ cell tumour (gct), gravid female, lactating adenoma, benign mature cystic teratoma

## Abstract

Mature teratoma is a benign germ cell tumor, histologically comprising components from mesoderm, ectoderm, and endoderm layer tissue. Here, we report a rare case of lactating adenoma arising from mature teratoma of the ovary in a pregnant female. To the best of our knowledge, only four cases of lactating adenoma arising from ovarian teratoma have been reported in the literature so far. This case is the fifth case reported worldwide, and the first case report with dual rare findings - choroidal plexus and lactating adenoma of mammary tissue in benign mature cystic teratoma. This is the second case report which uses immunohistochemical (IHC) markers to confirm the diagnosis. Grossly, the cystic structure was measuring 10x7x5cm. The cut surface revealed mixed solid and cystic areas filled with pultaceous material admixed with hair. Microscopy showed an ovarian cyst lined by stratified squamous epithelium with underlying sebaceous glands, apocrine acini, fatty tissue, smooth muscle, and glial tissues. Also noted mammary tissue composed of proliferating hyperplastic acini with central dilated ducts filled with eosinophilic secretions arranged in lobules. Immunohistochemistry with estrogen receptor (ER) and progesterone receptor (PR) showed luminal and ductal positivity. Strong expression of IHC markers such as p63 and pan-cytokeratin (pan-CK) was noted in myoepithelial cells and luminal cells respectively. Thus, confirming it as mammary tissue with hyperplastic ducts and acini.

## Introduction

Mature teratoma is an ovarian germ cell tumor consisting of mature tissues originating from two or three germ cell layers (ectoderm, endoderm, mesoderm). This is the most prevalent ovarian germ cell tumor, especially in young females in the reproductive age group. It accounts for 69% of germ cell tumors and 95% of teratomas [1]. The ectodermal derivative includes skin, sebaceous glands, hair follicles, and neural tissue, mainly glial tissues. Mesoderm derivatives such as bone, teeth, cartilage and smooth muscles, and endodermal structures such as gastrointestinal, respiratory, or bronchial epithelium, thyroid and salivary glands can be found. Despite being a benign tumor, older individuals rarely experience malignant transformation, which most of the time manifests as squamous cell carcinoma [2] or thyroid carcinoma [3]. We present the histological and immunohistochemical (IHC) findings of a sporadic case of lactating adenoma in a mature ovarian teratoma.

## Case presentation

**Figure 1:**
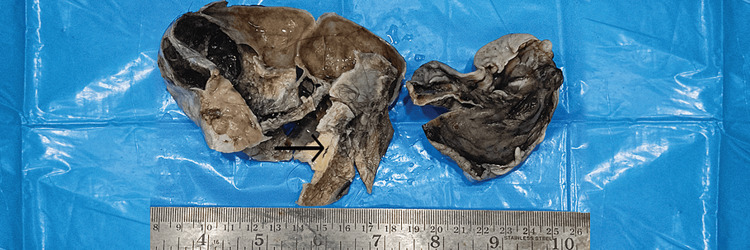
The gross image of ovarian cyst with cut surface showing pultaceous material with tufts of hair (arrow mark).

**Figure 2:**
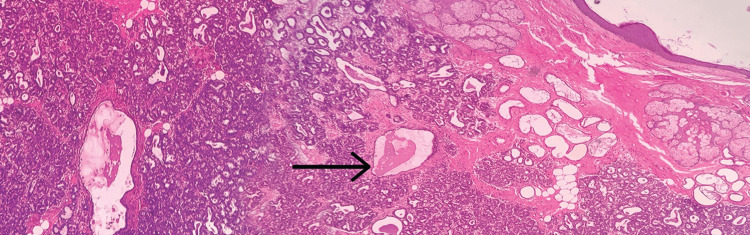
Microscopic examination Cyst wall lined by stratified squamous epithelium and pilosebaceous units. Mammary tissue with central dilated duct filled with secretions (arrow mark), 10x view (hematoxylin and eosin (H&E) stain).

**Figure 3:**
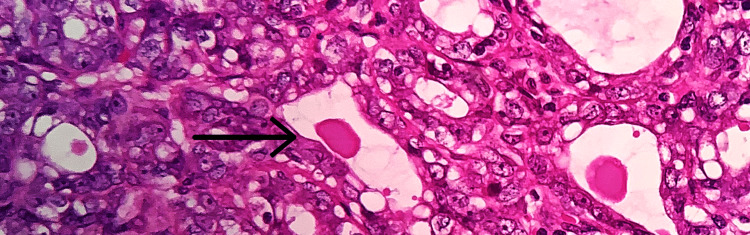
Microscopic examination Hyperplastic acini lined by cuboidal epithelium with round nuclei (arrow mark), 40x view (hematoxylin and eosin (H&E) stain).

**Figure 4:**
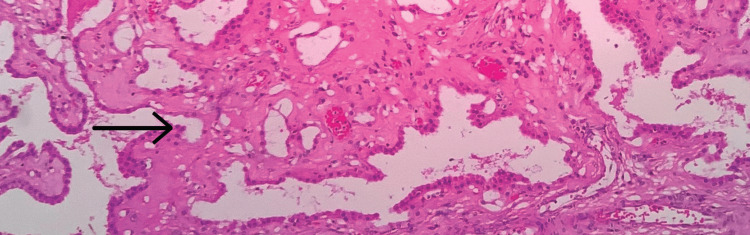
Microscopic examination Glial tissues with choroidal plexus (arrow mark), 40x view (hematoxylin and eosin (H&E) stain).

**Figure 5:**
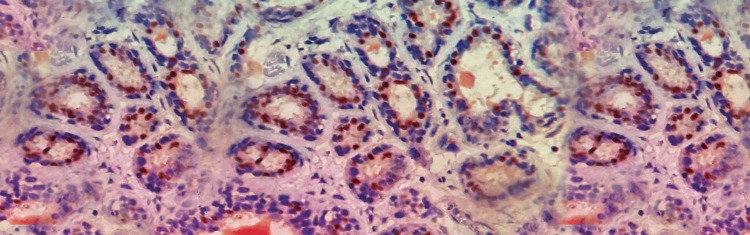
Immunohistochemical examination of ER The immunohistochemical marker estrogen receptor (ER) shows strong nuclear positivity in luminal cells of ducts in mammary tissue (40x view).

**Figure 6:**
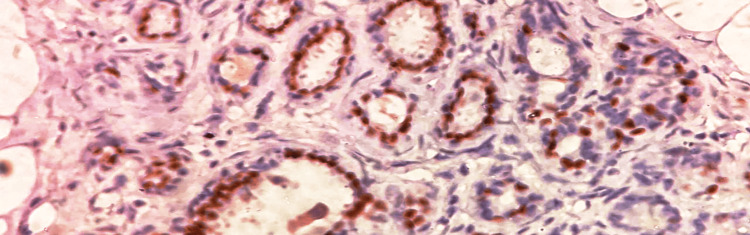
Immunohistochemical examination of PR The immunohistochemical marker progesterone receptor (PR) shows strong nuclear positivity in the luminal epithelial cells of ducts in mammary tissue (40x view).

**Figure 7:**
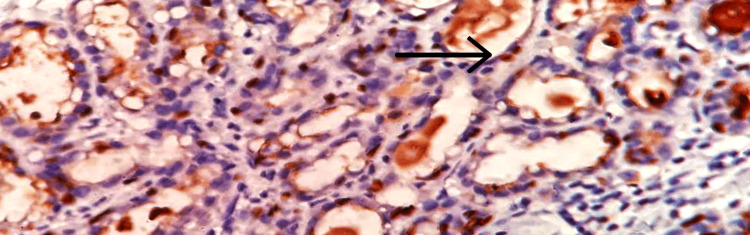
Immunohistochemical examination of p63 The immunohistochemical marker p63 shows positivity in the outer basal/myoepithelial cells (arrow mark) of the mammary tissue (40x view).

**Figure 8:**
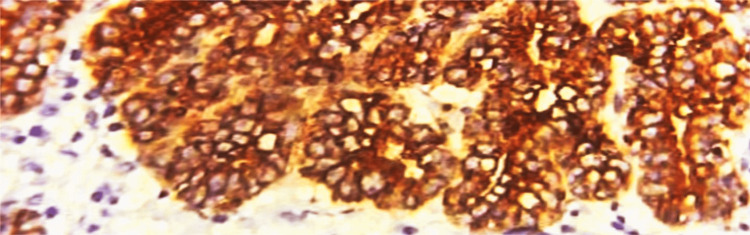
Immunohistochemical examination of pan-CK The immunohistochemical marker pan-cytokeratin (pan-CK) demonstrates diffuse strong positivity in the outer myoepithelial cells of the ducts in mammary tissue (40x view).

A 35-year-old female primigravida at 39 weeks of gestational age presented for the first time to the hospital with decreased fetal movements. The patient was admitted, vitals were checked and within normal limits, and then cardiotocography monitoring was started, and fetal distress was detected. The patient was immediately taken for an emergency lower-segment caesarian section. Intraoperatively, the right-sided complex ovarian cyst was noted on the table and removed. No antenatal scan was done before. Grossly, the cystic structure measured 10x7x5 cm (Figure 1). The cut surface revealed mixed solid and cystic areas filled with pultaceous material admixed with hair. Microscopy shows an ovarian cyst wall lined by stratified squamous epithelium with underlying sebaceous glands, apocrine acini, fatty tissue, smooth muscle, and glial tissues (Figure 2). Also noted is mammary tissue composed of hyperplastic acini with central dilated ducts filled with eosinophilic secretions arranged in multiple lobules (Figure 3). The hyperplastic acini are lined by one to two layers of low columnar epithelium with round nuclei and inconspicuous nucleoli (Figure 3). Secretory vacuoles and luminal secretions were also noted. We also noted glial tissue with choroidal plexus (Figure 4) in a few sections. Immunohistochemistry with estrogen receptor (ER) (Figure 5) and progesterone receptor (PR) (Figure 6) showed luminal and ductal nuclear positivity. Strong expression of IHC markers such as p63 (Figure 7) and pan-cytokeratin (pan-CK) (Figure 8) was noted in the myoepithelial cells and luminal cells, respectively. Hence, we confirmed the diagnosis as mature cystic teratoma with lactating mammary adenoma. Post-operatively, the patient was asymptomatic and on regular follow-up. No recurrence has been reported till now.

## Discussion

To the best of our knowledge, only four cases of lactating adenoma arising from ovarian teratoma have been reported in the literature so far [4-7]. This is the fifth case reported worldwide and the first case report with two rare findings: choroidal plexus and lactating adenoma of mammary tissue in benign mature cystic teratoma. This is the second case report, which uses IHC markers to confirm the diagnosis. The majority of teratomas contain tiny foci of the intestinal or colonic epithelium [8]. Tsubura et al. reported a similar case in a 30-year-old Japanese lady who is primigravida and delivered at 37 weeks of gestational age [4]. Ulirsch et al. reported a case of teratoma with neurogenic crest and lactating breast tissue in a young gravid female [5]. Although ovarian teratoma frequently involves breast tissue, it is rare for secondary neoplasms, such as lactational adenoma, to form from it. This can make diagnosis harder, especially since teratoma can affect a lot of different tissues. Kato et al. described a case of lactating adenoma-like breast tissue that looked like a benign, mature ovarian teratoma [7]. They found that the glandular epithelium expressed the IHC marker GATA-3.

In this case, the grossly well-circumscribed nature of the lesion and its correlation with a history of current or recent pregnancy will be helpful clues. IHC markers for breast tissue, such as ER and PR, both epithelial markers, exhibit strong nuclear positivity [9,10]. Additionally, p63 and pan-CK, specific myoepithelial markers associated with lactating adenoma, also show positive results [10]. This confirms the diagnosis of a lactating adenoma arising in a mature teratoma.

In similar studies, Moghaddam et al. and Venizelos et al. reported sebaceous carcinoma arising in mature teratoma [2] and [11]. In a young woman, Uzum et al. reported thyroid carcinoma arising from mature teratomas [3]. Tang et al. reported mucinous cystadenoma in mature ovarian teratoma [12]. Hackethal et al. and Dos Santos et al. reported squamous cell carcinoma in mature cystic teratomas [13-15]. Harms et al. say that conventional somatic malignancies like carcinoma, sarcoma, and leukemia can also arise from teratoma [16]. The neoplastic transformation process accounts for only 1-2% of mature dermoid cyst cases [17]. Around 2-3% of teratomas presenting in the pediatric age group have shown malignant transformation [18]. According to Trabzonlu et al., any ovarian teratoma in postmenopausal women should raise suspicions of malignancy [19]. Though secondary neoplasm arising in mature teratoma is a rare manifestation, it is of utmost importance for therapeutic and prognostic purposes. Surgical excision is the preferred treatment; incomplete removal of the cyst poses a higher risk of relapse [20].

According to Blackwell et al., neural components are present in just 37% of mature teratomas, while the choroidal plexus is only present in 2% of instances. Therefore, the observation of choroidal plexus in a mature teratoma, as in this case, is unusual [21].

## Conclusions

It is sporadic for lactational adenoma to develop in teratoma. Thorough grossing and careful histopathological examination, with the aid of IHC markers and knowledge of the spectrum of lesions that might develop in a teratoma, can lead to a correct diagnosis. Secondary neoplasms arising from teratoma, although rare, should be kept in mind while examining all cases of teratoma.
